# Smart Electronic Laboratory Notebooks for the NIST Research Environment

**DOI:** 10.6028/jres.120.018

**Published:** 2015-12-02

**Authors:** Richard S. Gates, Mark J. McLean, William A. Osborn

**Affiliations:** National Institute of Standards and Technology, Gaithersburg, MD 20899

**Keywords:** cloud, collaboration, computing, ELN, electronic, laboratory, notebook, SELN, tablet

## Abstract

Laboratory notebooks have been a staple of scientific research for centuries for organizing and documenting ideas and experiments. Modern laboratories are increasingly reliant on electronic data collection and analysis, so it seems inevitable that the digital revolution should come to the ordinary laboratory notebook. The most important aspect of this transition is to make the shift as comfortable and intuitive as possible, so that the creative process that is the hallmark of scientific investigation and engineering achievement is maintained, and ideally enhanced. The smart electronic laboratory notebooks described in this paper represent a paradigm shift from the old pen and paper style notebooks and provide a host of powerful operational and documentation capabilities in an intuitive format that is available anywhere at any time.

## 1. Introduction

**Fig. 1:**
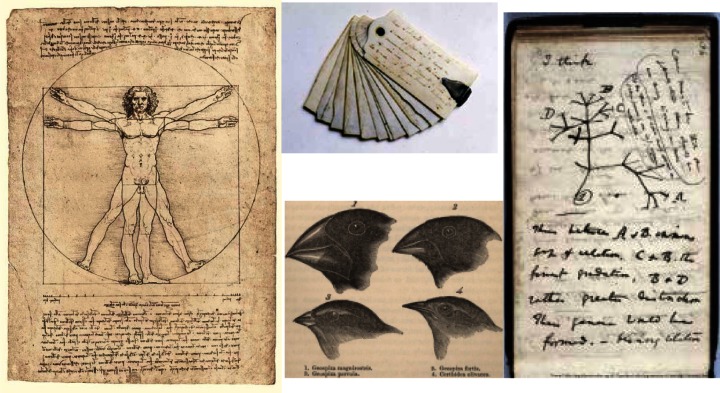
Examples of early lab notebooks: Leonardo da Vinci’s Vitruvian Man illustration, Thomas Jefferson’s portable/re-writable ivory leaf note pad, Darwin’s first rough sketch of the evolutionary tree concept, and his illustrations of Galapagos Finches.

Laboratory notebooks have been used for centuries to document and archive the thoughts and work of inventors, scientists, and engineers. Even before the invention of paper, Archimedes (circa 287 BC – 212 BC) [1] made notes and drawings of his mathematical discoveries and mechanical inventions on parchment made from animal skins. Leonardo da Vinci (1452–1519) [2] encapsulated perhaps some of the best known early combinations of experimental documentary concepts when he put pen to paper to combine his creativity and artistry in a format that has endured well beyond his lifetime (see Fig. 1). He even employed a degree of data encryption in his mirror writing, to provide a measure of privacy. Benjamin Franklin (1706–1790) and Thomas Jefferson (1743–1826) not only kept notebooks on their ideas and interests but they utilized a portable version of a memo pad made from thin ivory leaves, fastened together at one end so they could be fanned out for use [3]. These were re-writable; therefore, notes had to be ultimately transcribed to permanent notebooks for archival purposes, but the portability aspect was a key feature. Charles Darwin (1809–1882) [4] is another familiar example of someone who combined artistry with documentation to provide compelling evidence that led to his theory of evolution. During his long (1831–1836) voyage on the Beagle, he used fourteen different notebooks on his extensive field excursions, with a separate notebook dedicated to each major location. Later, prolific inventors like Thomas Edison (1847–1931) [5] made extensive use of laboratory notebooks to document exploration into a wide variety of fields. This was mainly to organize and document information to serve as a legal instrument for the development and protection of patents, so an audit trail was an important feature. But pen and paper is a static and limited medium. Enter the computer age and the internet where data, text, and images can be stored and transported as electrons and we have a new paradigm: the electronic laboratory notebook (ELN).

Early versions of ELNs were typically software features within main computers (mainframes, desktops) and associated with data collection from an instrument or group of instruments in a laboratory. They were often referred to as laboratory information systems (LIS), laboratory management systems (LMS), or laboratory information management systems (LIMS) [6]. They were used to collect, analyze, and organize data and even streamline the output of recently collected data into reports that could be stored electronically, and printed and sent to clients or colleagues. These programs were usually highly specific to an instrument or field and proprietary to the instrument vendor [7]. Later, more generic versions attempted to be more encompassing and universal for a variety of documentation and data management tasks for the bench scientist [8–10]. The development of portable computers and Ethernet networking provided a measure of transportability and access to the process that added value to the concept. The Internet, WiFi, and the World Wide Web further enhanced and accelerated that evolution. The electronic laboratory notebook described in this paper^1^ is a modern solution that closely mimics the form factor, portability, and convenience of the classic paper lab notebook. It has been enabled by the evolution and convergence of several key technologies involving hardware, software, networking, and cloud-based infrastructure, to allow a seamless and powerful tool that could be used by a wide variety of scientists and engineers in many disciplines. The solution documented here is only one of many that could be used. It was selected and optimized based on the research needs and resources of the authors along with the externally applied constraints of the information technology (IT) infrastructure of the National Institute of Standards and Technology (NIST). The solution is termed a “smart” electronic laboratory notebook (SELN) because it also incorporates several advanced sensor, communications, and documentation features (e.g., cameras), more familiar to users of “smartphones” than lab notebook users.

## 2. Guiding Concepts and Capabilities

This study began in early 2013 as a series of desires in a wish list. The authors conduct scientific and engineering research in several different laboratories in buildings located throughout the NIST main campus, including one cleanroom suite located 12 m underground, and the NIST Nanofab facility. Lab notebooks are a staple of conducting experiments every day, but the increasing sophistication of those experiments requires more and more information at your fingertips. The contents of a single paper laboratory notebook were not enough, and often copies of reference information and data printouts had to be stuffed into the notebooks as supplements. Coupling that to the use of clean room facilities (both our laboratories and the NIST Nanofab), where special cleanroom notebooks are required, and bringing in additional data required planning ahead to print it on special cleanroom paper. Other, more practical concerns included visual documenting capabilities (e.g., correct wiring for repairing an instrument is simplified when a smartphone camera is handy), web access, communication, and data sharing. Eventually, a cohesive set of guiding concepts and capabilities for an ideal SELN emerged. These are summarized in Table 1.

Since scientists are accustomed to carrying laboratory notebooks around, this approximate form factor (US Letter = 8 ½” × 11”) was a reasonable starting point in terms of general size. Portability requires a lightweight device (under 1 kg), capable of sustained performance while unplugged and powerful enough to handle the significant amount of computation, graphics, and Input/Output required in a scientific laboratory environment. The amount and type of interaction with the device also requires a relatively large, high-resolution screen which further strains the long battery life requirement. The software and interface should be intuitive and operate in a manner that is simple and familiar. The current generations of touchscreen tablet devices and their accompanying mature operating systems satisfy these criteria. Addition of a robust note-taking software application or package with familiar organization mimicking the organizational look and feel of paper (notebooks, folders, pages) completes this need. Our research team works in both a local cleanroom laboratory and the NIST Nanofab; therefore, cleanroom compatibility is an important capability. This requires smooth, easily wiped surfaces for particulate control during entry to the clean room laboratories. Device materials and surface textures should not generate or easily trap particles. Care should also be taken with moving parts like cooling fans that may inadvertently produce streams of particles at exit ports during operation. The creative concept is meant to inspire innovative thought and imagination for the scientist/engineer user by providing an easy to use, flexible, environment to organize their notebook. This enables better problem solving and documentation. With paper systems, scientists sometimes tape data printouts, pictures, and tables into their lab notebooks. For electronic versions, the desire was to allow maximum flexibility for multiple input/output and storage formats to handle handwritten drawings and notes, text, images, data files, and audio. Stylus input can take advantage of artistic talents. Audio recording or speech-to-text could be convenient for taking a note when hands are occupied, or video recording could provide the ultimate visual documentation for an important experiment. For the authors’ research team, collaboration through resource sharing was a driving consideration. Working in a microfabrication facility is a complex, expensive endeavor and the ability to share resources and information on tools, processes and results is extremely valuable. With a small team, trust and etiquette are manageable but with a larger community, the ability to restrict access or editing capability might be required. The “Live” concept of all your information going with you at all times is a powerful feature made possible by the combination of wireless networking (WiFi) and cloud computing. Finally the “Smart” concept is just a realization of what additional features a modern electronic lab notebook system can have when it is allowed to access the myriad of sensor technologies of the digital age. Cameras for both picture and video capture open up a wealth of documentation capabilities. Communication can be as simple as e-mail or text messaging, or as full featured as video conferencing. Most tablet devices are starting to incorporate sensors like accelerometers, gyroscopes, and magnetometers, for specialized capabilities that are still being fully developed. Smart can also mean help to organize and retrieve all the saved information through tagging and indexing since one of the hazards of being able to collect large amounts of information is finding it when you need it. There are also many practical IT constraints imposed that are required for effective, safe, and secure implementation. Additional constraints imposed by NIST IT security requirements include secure BIOS, full disk encryption, firewall, password protection, domain authentication, and antivirus protection.

**Fig. 2:**
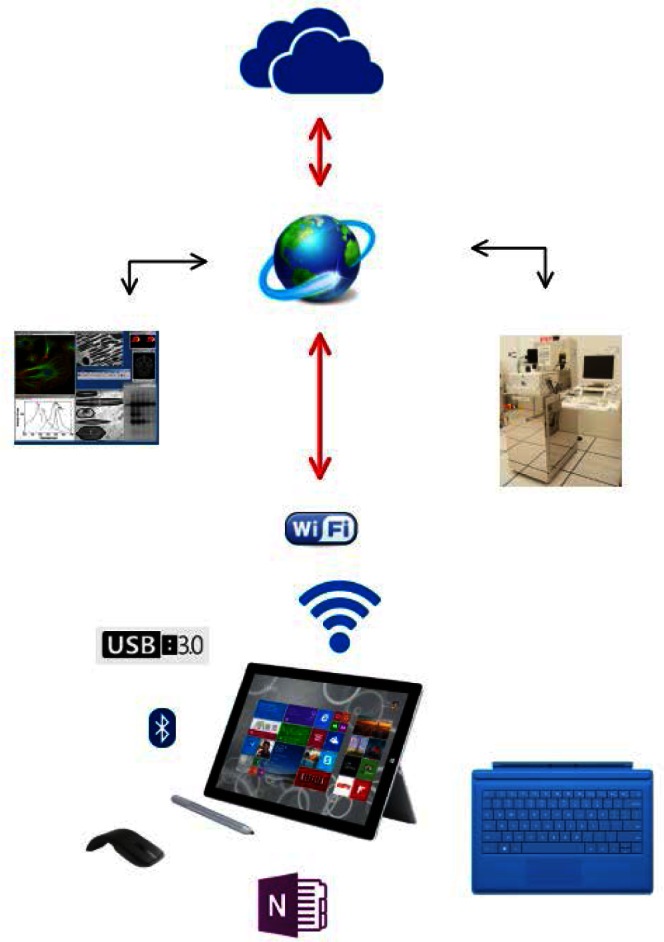
The smart electronic laboratory notebook (SELN).

The wish list that makes up the SELN concept can be partially visualized in the composite schematic shown in Fig. 2 but the real complexity is hidden behind invisible but critical issues of compatibility, protocols, standards, security, and infrastructure for both hardware and software.

Why now? Many of these individual rudimentary concepts and features described may have been around for a while but the main reason it has been brought together in the system described in this paper is that technology today is advancing rapidly on many fronts and in many ways. The combination of evolution and maturation of certain technologies and their convergence has given rise to this opportunity only recently. The key technologies include:

- Hardware (CPU, battery, touchscreen)
- Software (OS, Notes)
- Network Infrastructure (WiFi)
- Cloud Computing & Storage (Infrastructure & software)
- Cleanroom access and tool management system (via Web)

Within hardware technology the important components include the central processing unit (CPU), battery, and touchscreen. The constant evolution of CPU speed and power are familiar to most consumers who see “faster, smaller, and cheaper” as the mantra of computer technology. What is equally important though is the evolution of low power consumption CPUs combined with better battery energy storage solutions. Touchscreen technology also has competing interests of power consumption and size, so tradeoffs are often required. Software plays important roles in both the operating system, and note taking and organizing software. Operating systems are becoming more intuitive as touchscreen devices are more ubiquitous and the human-device interface becomes more useable and familiar. Note taking software has evolved past the simple notepad and sticky-note applications to full featured programs like Evernote and OneNote. WiFi has cut the physical network cord through an alphabet soup of 802.11 standards and now this network infrastructure provides a flow of information that is fast, secure, and convenient. Cloud computing as implemented by both hardware (servers) and software capable of utilizing it, provides a transparent medium where information available anywhere at any time is a realistic expectation. Finally, the recent development of a web-based facility access and tool management system in the NIST Nanofab was a major driving force for our research team, all of whom are heavy users of that facility.

## 3. Device and Software Selections

There are many hardware options available today that are computationally powerful touchscreen devices with a suitable form factor (10” screen or greater) and sufficient battery life to handle an extended session of several hours in a laboratory or clean room without being plugged in. The authors purchased and evaluated several devices including tablets, ultrabooks, and hybrids (essentially tablets with docks mainly used together but detachable for the cleanroom environment). After using these devices extensively, the team compared their experiences to provide feedback and recommend several important hardware features for an optimal device for further pilot study deployment. This included a rear facing camera (in addition to front facing) for visual documentation, a good stylus for more detailed screen drawing, and a cleanroom-compatible (or detachable) keyboard (w/integrated trackpad or mouse) for more versatile documentation and manipulation when necessary. A USB (3.0) port was also important for transferring data from an instrument in the lab to the SELN through a thumb drive. The current system is a 12” touchscreen Microsoft Surface Pro 3 tablet with an attachable keyboard/cover, and a Bluetooth 4.0 enabled mouse and stylus. It runs on Windows 8.1 Pro and has 802.11 ac WiFi capability.

There are many different software packages and applications available for taking notes electronically on a variety of computing devices [11]. For the organization/note-taking software option of this study the team utilized Microsoft OneNote. Logistically, this was a good fit since this program was already available across NIST as part of the Microsoft Office 365 site-wide subscription rollout in 2013. The OneNote program represents a mature version of a free-form note taking architecture that can be shared among people across multiple platforms. It has evolved considerably since it was first introduced in 2003 and as tablets and cloud computing have evolved, it has kept pace to provide a well-integrated, intuitive interface. Hardware – software integration also helps strengthen the overall user experience. For example, the stylus for the Surface Pro 3 has a button that automatically brings up the OneNote program on the screen and dedicated buttons to enable erasing and “right mouse button” functions. Clipping applets are also available to automatically send clips of screen images to OneNote pages. Finally, the Surface Pro 3 has both handwriting recognition and the ability to personalize it for your own particular handwriting, along with palm blocking technology, so resting your palm on the touchscreen does not interfere with writing or drawing.

**Fig. 3:**
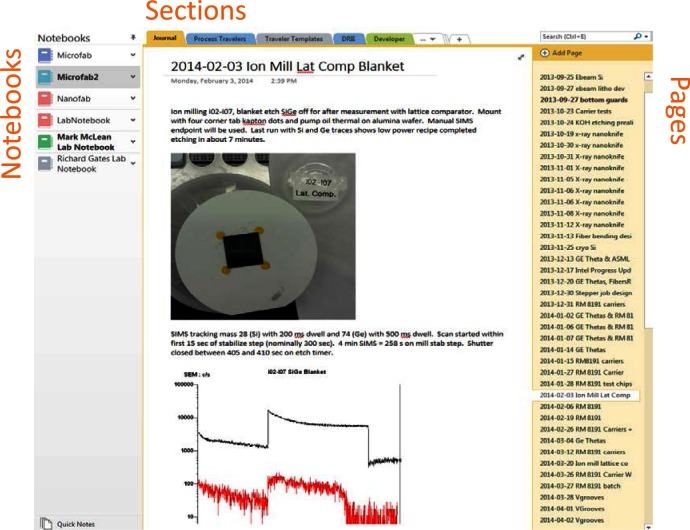
OneNote organizational layout of the nested Notebook/Section/Page hierarchy with the contents of the selected page displayed in the center.

OneNote is an example of a flexible information gathering program with an intuitive organizational structure based on familiar “paper” concepts of notebooks, sections, and pages as shown in Fig. 3. The terms have a nested hierarchical structure such that notebooks can contain many sections, sections can contain many pages, and each page is virtually unbounded in both x and y dimensions. The application itself can have a slightly different “look and feel” depending on the platform and version, since it is available in standalone, web browser-based, and application-based versions specific to the particular operating system and device. Typically, the individual notebooks are stacked in a column on the left side of the screen, the sections are represented as a series of tabs across the top, and the pages are represented as a stacked column of page title entries on the right. Navigation is fairly straightforward: click on a notebook to see its sections, click on a specific section to see its pages, and click on a particular page title to see its contents displayed in the center of the screen. Sections themselves offer the ability to “group” under topics (click to expand a group into its constituent tabs) and page titles have the ability to indent two levels for further simple visual organization.

Each page in OneNote is free format and supports any combination of handwriting, drawing, text, tables, images, and attachment of any manner of inserted files and hyperlinks. Items can be tagged and pages are searchable. Optical character recognition (OCR) even allows most indexing functions to work with handwriting.

Notebooks are automatically saved in real-time and can be stored locally or on the cloud. Once on the cloud, they are still private but now available to the owner on any other platform. They can also be shared with individuals (either as read-only or editable) by extending an e-mail invitation using the sharing function within the OneNote program. Pages (or even entire notebooks) can be printed, e-mailed, or exported in several formats, including. xps,. doc, and. pdf if needed.

Notebooks can be defined for a variety of different functions or tasks, and customized accordingly. Each team member for example has a personally identified Lab Notebook shared with the rest of the team. Other shared resource can be appropriately named for the topic. For example, a shared “Nanofab” notebook is a common resource where everyone contributes to tool processing and performance information of mutual interest. Other collaborative notebooks might exist for a paper or perhaps a training document for sharing with others beyond the team. Beyond these uses, each team member has subject or project-based (e.g., Standard Reference Materials, Additive Manufacturing) or role-based (e.g., Management), notebooks that may be local to a particular PC or shared only with themselves on different platforms. The organization and development of the content of this paper was facilitated by creating a SELN notebook, with appropriate sections and pages to collect resources and document ideas and concepts.

SELNs can provide a seamless, transparent, organization and order to the multidimensional aspects of research in the laboratory in a way that paper notebooks cannot. Unless separate paper notebooks are used for each project, a paper lab notebook contains a linear chronological record of research on many experiments and topics. Researchers often leave a couple of pages at the beginning of the bound paper notebook with the intent of coming back later to build a table of contents for later quick reference. Despite good intentions, many times this is never completed. OneNote maintains a chronological order of all entries but it is entirely in the background. The foreground, the graphical interface actually presented to the user, is a friendlier organization of topics in the manner selected by the user. In this sense, the table of contents is generated continuously and simply through the user’s use of tabs and pages within the electronic Notebook. A change in organization is accomplished with a simple drag and drop of sections or pages while any underlying chronology is maintained transparently in the background.

One somewhat unusual aspect of OneNote is the lack of a save button. This is because it constantly saves and synchronizes in the background whenever possible. OneNote also continues to function when off-network. If changes are made to a notebook while not connected to the cloud, the program will wait until it is reconnected to synchronize. Notebooks that are resident on the cloud are also intrinsically backed up. The history menu provides a simple means of reviewing recent edits to any portion of a notebook and a page version history allows complete access to previous versions of a page which can then be restored if desired. Shared versions contain information on edits by all contributors, consequently, a reasonably simple audit trail exists for all edits. While this trail is useful, it can be disabled by someone with write privileges, so it may not be as legally rigorous for patent or regulatory compliance (e.g., FDA Title 21, CFR Part 11).

## 4. Examples of SELN Use

**Fig. 4:**
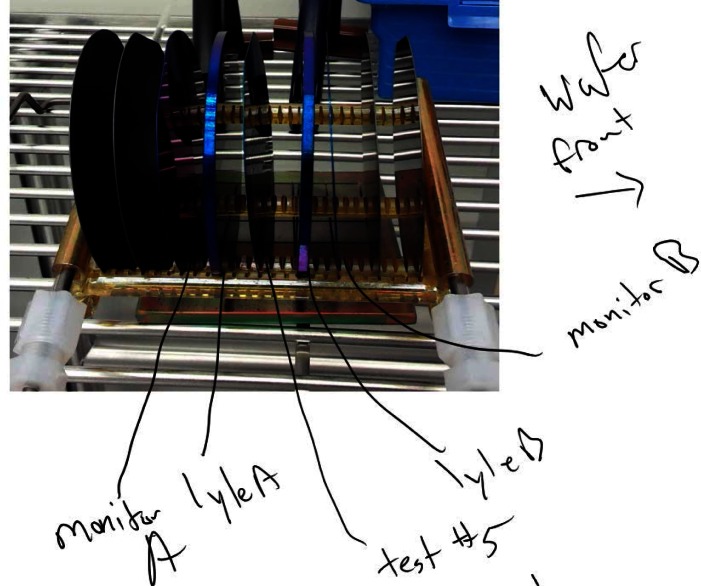
Typical experiment documentation capability of the SELN combining the rear facing camera and hand annotation.

The most common and straightforward use of a SELN is as a journal to document each experiment as it is being conducted. Pages are typically listed in chronological order (just like a paper lab notebook) with Content however is much more powerful. For example, the picture in Fig. 4 was taken in the NIST Nanofab using the SELN device’s rear facing camera, pasted right into a journal entry page and then hand annotated using a scribe. The annotation identifies the contents of the tray of Si wafer samples from a tube furnace that were later analyzed for film thickness. Variations in experiment results can then be traced back to changes in the tool performance (or perhaps location in the furnace tray) that might affect subsequent users, so sharing of this information is valuable to all involved.

A second important use of SELNs is for collaboration and sharing - posting information useful to all members of the team. One team member might post a calibration curve for an instrument that another member could use the following day. A user might update an inventory list the team has, and when someone is looking for a part, this information is always updated and available. Another way of sharing information is through administrative, read only notebooks. This would be a valuable shared resource for clients in many multi-user environments like a Nanofab. There are many available tools that must be calibrated and qualified using standard recipes. This data could be easily shared, so users could check for any process trends against their own test wafers before committing their valuable research samples.

SELNs also provide a more convenient means of conducting research in the NIST Nanofab. Since 2014, access to the Nanofab and process tools was available to users through a new web browser based tool developed at NIST known as NEMO (Nanofab Equipment Management Operation) [12]. NEMO is required for important tasks dealing with tool reservations (checking, making, and deleting), tool operation (status, enable and disable), and to check to see who is currently working in the Nanofab (both staff and users). Cleanroom entry, reservations, and tool usage are tied into billing applications and tools are interlocked to prohibit tool use without first enabling them through NEMO. Reservations can be accessed from anywhere and are often made from outside the Nanofab from an office desktop for example. For actual tool use within the Nanofab, NEMO is usually accessed from several hardwired workstations dispersed locally throughout the facility. Since these systems are also used to access e-mail and web-based resources, there can be a bit of a backlog when the Nanofab is being heavily used. The SELN allows wireless, authenticated access to the NEMO web application from anywhere, making use of the tools simpler and more efficient, especially from within the Nanofab where the user can sit in front of the tool being used and have complete control over its immediate access. Having an SELN available during the frequent down times in the Nanofab (e.g., waiting for processes to run or waiting for tools to open up) also makes efficient use of free time for communication, computation, or analysis of data.

SELNs are also fully functional computing devices with enough computing power to run most software programs. This provides a degree of problem solving freedom that has to be experienced to be appreciated. A recent example in our labs involved the need to secure an optical lens component at a particular orientation to an instrument in the lab. A few measurements directly input to a CAD program using the SELN and then wirelessly sent to a 3D printer queue and the part was on its way.

Having easy access to many electronic resources everywhere at all times is very empowering. From e-mail, web information, laboratory temperature and humidity sensors, the NIST library, to messaging, video conferencing and even your office PC (through remote access).

## 5. Discussion

The best features of a SELN are personal to each user since they are so flexible and adaptable that customization is easy and each individual has their favorite uses. The authors all work in the lab; therefore, the most useful common feature of the SELNs was the rear facing camera. That, combined with hand annotation has saved a lot of time with issues like equipment repair and quick and detailed experiment documentation. The camera also provides the opportunity to couple with image recognition software in a variety of ways and deliver powerful capabilities, just as it does in smartphones. Document scanning and optical character recognition makes later cutting, pasting, and reorganizing of subsets of information more streamlined. Laboratory sample, chemical, and equipment tracking and organizing is made more powerful and efficient using barcode and quick response (QR) codes.

Sometimes even the more mundane aspects make the whole SELN experience more seamless and quietly powerful. Web access, for example, comes in many forms of utility that shape the day’s experiments. Several of our labs have temperature/humidity sensors that can be accessed remotely via the web, so a quick check on the lab before and during an experiment makes sure there are no environmental issues that need to be addressed. Since some labs are buried 12 m underground it is quite easy to lose track of time or what is happening outside. A quick check of the weather app before running a sensitive experiment might reveal an approaching line of thunderstorms that would advise delaying the experiment.

In addition to understanding how well SELNs can be used at NIST, the pilot study has pointed out several issues in the IT infrastructure that could be addressed to provide a more seamless SELN experience. Making sure WiFi is available at all locations, from office to lab, for each research team is an obvious need. Expanding technical support to include the newest tablet hardware and software is needed to streamline the rollout of the SELN to more researchers throughout NIST.

The initial pilot study of 3 participants was expanded to 6 in the latter half of 2014 and is destined for a larger (≈100) series of incremental rollouts across our operating unit (Materials Measurement Laboratory) through 2015. To assist in this, a demo OneNote notebook has been developed and made available to new participants at NIST to provide a bit of an introduction into the capabilities and best practices of the SELN, so people can self-train or explore the concept as the pilot expands.

Another important area where the SELN may play a key role is the recent US Government policies and requirements for open data [13] and data management plans [14]. These new principles, guiding the management of digital data began in 2013 and are being implemented across the scientific communities in the Federal Government. The combination of organizational capabilities and export options in the SELN described in this paper may offer a pathway for better data management and data curation.

For some scientists, the laboratory could be “in the field” where cellular service and GPS might be utilized to provide additional functionality. WiFi hotspots are becoming common on smartphones, so even tablets that do not have cellphone capability can be linked in with a smartphone. This might be useful to archaeologists, geologists, marine biologists, or other field scientists.

## 6. Future Vision

In using the SELN concept in a variety of ways within the pilot project team at NIST, it becomes apparent not only how useful it would be across a wider NIST audience but how future advances may open whole new areas of utility as new “smart” features get smarter. For example, the area of wearable sensors has exploded recently with watches [15] and sports bands [16] able to measure steps, heart rate, and even location, and buffer and communicate this information to a nearby smartphone (or SELN) via Bluetooth. One can imagine the day when lab scientists might wear a “safety” band capable of measuring local medical diagnostic and environmental information. Monitoring of heart rate, temperature, humidity, oxygen concentration and perhaps the presence of toxic chemicals or radiation could be linked to the SELN through Bluetooth. An irregular reading might trigger an audio warning to the scientist and immediately alert nearby staff or emergency personnel of the potential danger, so assistance could be rendered. Two-way communication could be used to augment the “buddy system” often used for dangerous tasks in many labs. These small satellite sensor devices could also take the form of pens or badges that could be simply clipped to a pocket or lapel.

The other major relevant IT technology trend is the internet of things in which device interconnectivity may offer new capabilities not even thought of yet. In the smaller, more specialized, and secure environment of the laboratory or larger research facility it may manifest itself as the intranet (or VPN) of things (instruments) where the SELN may play a critical role as the personal, portable, collection and access point where data is organized, analyzed, and displayed anywhere at any time.

The rapid and continuous evolution and rollout of new hardware, software and human interface engineering shows little sign of slowing down. Siri is already available for iPad and Cortana is included in the Win10 version of tablets released in mid-2015. So-called “phablets” may be another potential SELN version for those requiring more portability. The future is right around the corner.

## 7. Conclusion

The time is right for a smart electronic version of the laboratory notebook to make its way into the mainstream NIST research environment and beyond. While there are many hardware form factors and versions capable of serving as an SELN, the version demonstrated in this paper represents a rather high end, powerful one, well suited for the wide range of activities of our pilot team. It provides a comfortable portability and connectivity crucial to its role, and could actually also serve as a desktop replacement for many who do not require extremely powerful computing resources.

The combination of large touchscreen, intuitive interface, powerful computing, and flexible and intuitive organizational software provides a firm foundation for collecting and organizing laboratory documentation as well as a wide variety of topical projects (from management to research). Since the SELN is linked to a wireless network and all information can reside on the cloud, access can be from anywhere at any time. Information can be shared, either with yourself on other devices, or with colleagues. Privileges can be adjusted to the level of trust desired to allow different degrees of collaboration. The upper end of the capability of this type of SELN resource is only limited by the creativity of the user and as technologies advance and infrastructures improve, will only become more essential.

## Figures and Tables

**Table 1:** Guiding concepts for the development of a smart electronic laboratory notebook (SELN) for the NIST research environment.

Guiding Concept	Capability	Details
Portable	Take with you anywhere	Hardware lightweight with long battery life
Intuitive	Touchscreen hardware. Operating System and application software capable of familiar use and organizational style	Graphical interface OS. Notebooks/Folders/Pages format style
Clean	Cleanroom compatible	Tablet style easily wiped down for cleanroom entry
Creative	Flexible & easy to use	Multiple formats: text, images, audio, video, handwritten notes, stylus
Collaborative	Resource sharing	Variable control for the “circle of trust”
Live	Information available anywhere at any time.	Wireless and Cloud
Smart	Features not normally associated with a lab notebook.	Camera, microphone, communications, sensors, web access, tagging and searchable indexing
Secure	NIST IT security constraints	Password protection, disk encryption, firewall, domain authentication, antivirus

## Footnotes

1 Certain commercial equipment, instruments, or materials are identified in this paper to adequately specify the experimental procedure. Such identification does not imply recommendation or endorsement by the National Institute of Standards and Technology, nor does it imply that the materials or equipment identified are necessarily the best available for the purpose.
